# Safety, Quality, and Acceptability of Contraceptive Subdermal Implant Provision by Community Health Extension Workers Versus Nurses and Midwives in Nigeria: Protocol for a Quasi-Experimental, Noninferiority Study

**DOI:** 10.2196/resprot.8721

**Published:** 2018-03-02

**Authors:** Kate Reiss, Suzanne Penfold, Olalere Alabi, Moazzam Ali, Kristen Hopkins, Thoai Dinh Ngo, Kingsley Odogwu, Megan Douthwaite, Onoriode Ezire, Uko Udoh, Effiom Effiom, Erik S Munroe

**Affiliations:** ^1^ Department of Population Health Faculty of Epidemiology and Population Health London School of Hygiene and Tropical Medicine London United Kingdom; ^2^ Evidence to Action Team Technical Services Department Marie Stopes International London United Kingdom; ^3^ Marie Stopes Nigeria Abuja Nigeria; ^4^ Department of Reproductive Health and Research World Health Organization Geneva Switzerland; ^5^ Independent Consultant London United Kingdom; ^6^ Poverty, Gender and Youth Programme Population Council New York, NY United States; ^7^ Palladium Group Abuja Nigeria; ^8^ Department of Health Planning, Research and Statistics Federal Ministry of Health Abuja Nigeria

**Keywords:** drug implants, contraceptive prevalence, contraception, delivery of health care, family planning services, task shifting, Nigeria, community health workers, long-acting reversible contraception

## Abstract

**Background:**

As part of its Family Planning 2020 commitment, the Nigerian government is aiming for a contraceptive prevalence rate of 36% by 2018, and in 2014, approved a policy to allow community health extension workers (CHEWs), in addition to doctors, nurses, and midwives, to provide contraceptive subdermal implants. There is a lack of rigorous evidence on the safety of long-acting reversible contraceptive provision, such as implants, among lower cadres of health providers.

**Objective:**

This study aimed to compare implant provision by CHEWs versus nurses and midwives up to 14 days post insertion.

**Methods:**

The quasi-experimental, noninferiority study will take place in public sector facilities in Kaduna and Ondo States. In each state, we will select 60 facilities, and from these, we will select a total of 30 nurses and midwives and 30 CHEWs to participate. Selected providers will be trained to provide implant services. Once trained, providers will recruit a minimum of 8125 women aged between 18 and 49 years who request and are eligible for an implant, following comprehensive family planning counseling. During implant insertion, providers will record data about the process and any adverse events, and 14 days post insertion, providers will ask 4410 clients about adverse events arising from the implant. Supervisors will observe 792 implant insertions to assess service provision quality and ask clients about their satisfaction with the procedure. We will conclude noninferiority if the CI for the difference in the proportion of adverse events between CHEWs and nurses and midwives on the day of insertion or 14 days post insertion lies to the right of −2%.

**Results:**

In September and October 2015, we trained 60 CHEWs and a total of 60 nurses and midwives from 12 local government areas (LGAs) in Kaduna and 23 LGAs in Ondo. Recruitment took place between November 2015 and December 2016. Data analysis is being finalized, and results are expected in March 2018.

**Conclusions:**

The strength of this study is having a standard care (nurse and midwife provision) group with which CHEW provision can be compared. The intervention builds on existing training and supervision procedures, which increases the sustainability and scalability of CHEW implant provision. Important limitations include the lack of randomization due to nurses and midwives in Nigeria working in separate types of health care facilities compared with CHEWs, and that providers self-assess their own practices. It is unfeasible to observe all procedures independently, and observation may change practice. Although providers will be trained to conduct implant removals, the study time will be too short to reach the sample size required to make noninferiority comparisons for removals.

**Trial Registration:**

ClinicalTrials.gov NCT03088722; https://clinicaltrials.gov/ct2/show/NCT03088722 (Archived by WebCite at http://www.webcitation.org/6xIHImWvu)

## Introduction

In 2012, an estimated 222 million women had an unmet need for modern contraception [1]. Meeting this need would avert an estimated 218 million unintended pregnancies and 118,000 maternal deaths [1]. Nigeria has a large (191 million in 2017) and rapidly increasing population [2] and a high maternal mortality ratio, estimated to be 576 per 100,000 live births in 2013 [3]. In 2013, 15% of currently married women aged between 15 and 49 years were using some method of contraception, a figure considerably lower than the global (63%) and sub-Saharan Africa (27%) estimates for the same year, and 16% had an unmet need for contraception [3].

There are many inequities in contraceptive access, with lower levels of use among women in rural and remote areas, and among those who are poorer, less educated, and younger [4]. In Nigeria, contraceptive prevalence is just 9% in rural areas versus 27% in urban areas [3]. In addition, the most effective contraceptive methods are often the most difficult to access [5]. Long-acting reversible contraceptives (LARCs), such as intrauterine devices (IUD) and implants, are the most effective reversible methods of contraception, with failure rates of between 0.05% and 0.8% in the first year of use (contraceptive pills have a failure rate of 8%) [6]. LARCs also have lower discontinuation rates than short-term methods (such as the pill, injectable, and condom) [5] and generally have high user satisfaction [7]. In sub-Saharan Africa, unlike Asia and North America, short-term contraceptive methods dominate [8]. In Nigeria in 2013, 9.8% of currently married women were using a modern method of contraception, and most (7.1%) were using short-term methods; just 1.1% were using an IUD and 0.4% an implant [3].Contraceptive implants consist of flexible matchstick-sized rods, inserted below the skin on the nondominant upper arm. They release small amounts of progestin hormone to prevent pregnancy for 3 to 5 years [9]. Insertion is quick and does not require pelvic examinations or laboratory tests. Complications are rare (although may include infection at the insertion site, expulsion, or difficult removal), and once inserted, implants require no regular action by the user or health system [6].

Key barriers to LARC use include lack of availability (of method or skilled providers), perceived cost, and misperceptions about risks and benefits [5,10,11]. Low LARC availability has been driven by lack of trained providers and, in the case of implants, high commodity costs [12]. In recent years, costs have fallen [13] but low provider numbers remain: the African continent faced an estimated shortage of 4.2 million health care workers in 2013 [14]. Shortages are more acute in rural areas: whereas the national average number of doctors per 100,000 population in Nigeria was estimated to be 12 in 2007, in the more rural northwest and northeast, the ratio was only 4 [15]. Task-shifting or task-sharing is a strategy recommended by the World Health Organization (WHO) to address health worker shortages [16]. This involves expanding specific tasks, where appropriate, from highly qualified health workers to those with shorter training and fewer qualifications [17]. Task-sharing of implant services has been implemented in several African countries [12]. After a policy change allowed nurses, as well as physicians, to provide implants in Tanzania, insertion rates increased from around 10,000 per quarter in 2007 to more than 20,000 in 2009 [18], and in Ethiopia, 15,000 rural community health extension workers (CHEWs) have been trained to insert implants (removals are still handled by higher-level cadres) [12].

In 2012, Nigeria’s Honorable Minister of Health announced a goal to increase contraceptive prevalence to 36% by 2018 [19]. The priority activities are to train more health care workers to provide injectables, implants, and IUDs [19] and to change national policy to permit CHEW provision of implants [20]. CHEWs are health care staff who undergo a 36-month course in a training institution approved by the Community Health Practitioners Registration Board of Nigeria [20]. CHEWs may be located in larger urban health centers, with nurses and midwives, or at smaller health centers, working alone or with another CHEW. In a pre- and posttest pilot study conducted in Sokoto and Bauchi States in Nigeria to assess the feasibility of training CHEWs to provide implants [21], 166 CHEWs were trained for 2 to 3 weeks, and they inserted 3588 implants in 151 health facilities over 6 months. Most CHEWs achieved competency in implant insertions after insertions with 4 to 5 clients. Clinical observations revealed that CHEWs performed implant insertion tasks correctly at least 90% of the time for 16 out of 19 checklist items. The amount of information that CHEWs provided clients increased between baseline and end line, and over 99% of surveyed clients reported being satisfied with CHEWs’ services.

Making implants more widely available could reduce the unmet need and contribute toward achieving the ambitious 2020 contraceptive prevalence rate goals (the percentage of women of reproductive age who are married or in union and are currently using or whose sexual partner is currently using a modern method of contraception) set by many countries in Africa and elsewhere at the family planning (FP) summit in 2012 [22]. A systematic review of the effectiveness and safety of task-sharing for the delivery of injectable contraceptives, contraceptive implants, IUDs, tubal ligation, and vasectomy in low- and middle-income countries found little or no difference between cadres, but admitted that only limited conclusions could be drawn from the small number of eligible studies [23]. The conclusion of this review and the recent policy change in Nigeria mean that there is opportunity and need to evaluate the safety, quality, and acceptability of implant services provided by CHEWs in Nigeria.

## Methods

### Study Design, Aim, and Objectives

The aim of this quasi-experimental, noninferiority study was to compare insertion of contraceptive implants (Implanon Classic and Jadelle; Table 1) by CHEWs with nurses and midwives in Nigeria.

The primary objective was to compare the safety of implant insertions by CHEWs with that of midwives and nurses on the day of the procedure and up to 14 days post insertion. Secondary objectives were to: (1) compare the quality of implant insertions by CHEWs with that of midwives and nurses; (2) compare client acceptability of implant insertions by CHEWs with that of midwives and nurses; and (3) assess acceptability of CHEW provision of implants to other health staff, clinic managers, and other key individuals such as policy makers.

For the purpose of this study, *safety* refers to implant insertions that minimize risk and harm to service users. We hypothesize that implant provision by CHEWs will be as safe and of high quality as provision by existing cadres of implant providers. Safety will be assessed by comparing rates of any adverse events (ie, those with a minor, moderate, or severe impact on a woman’s health) at the time of the procedure, or up to 14 days post insertion (Table 2). As the study is evaluating the provider and not the method itself, method-related adverse events such as hormone-related changes to menstruation pattern, headaches, and nausea will not be recorded; neither will the study examine the effectiveness of the method.

We define *quality* as the degree to which a provider or facility meets certain objective and subjective levels of health care delivery standards. The term covers all aspects of clinical service provision such as correct insertion, infection prevention, and disposal procedures, as well as pre- and post counseling and taking a client-centered approach. We define *acceptability* as the level of satisfaction experienced with the service received (in the case of the client) and the level of satisfaction with this aspect of the job (in the case of the provider).

### Study Implementation

This study is a partnership between Marie Stopes International (MSI); Marie Stopes International Organisation Nigeria (MSION); the Federal Ministry of Health of Nigeria (FMOH); Kaduna and Ondo State Ministries of Health, Nigeria; the WHO’s Department of Reproductive Health and Research Geneva; and the University of Ibadan, Nigeria. Clinical training and research components will be conducted by MSI and MSION. MSION established its first clinic in Abuja in 2009 and now serves women in 25 states. Its mission is to provide reliable information to women about their FP options and to improve their access to FP methods. MSION is one of the only providers of LARCs in Nigeria. Services are delivered through clinics, mobile outreach teams, a social franchise network of private providers, and partnerships with government providers.

### Site and Provider Selection

The study will take place in public sector facilities in 2 Nigerian states, 1 in the north (Kaduna) and 1 in the south (Ondo). The north and south of Nigeria differ in religious beliefs, contraceptive prevalence rate, and availability of health care providers, making it necessary to generate evidence in each context. We will purposively select local government areas (LGAs) in Kaduna and Ondo States for study participation by excluding LGAs with overlapping interventions and, given the extensive supervision needs of the study, hard-to-reach LGAs.

Facilities will be eligible if they provide referral services on site or are located within 20 km of a referral facility, in case of adverse events resulting in the need to refer clients; if there is a provider interested in participating in the study who expects to be in the facility for the 12-month period of client recruitment; and if the facility has been providing FP in the previous 3 years and does not currently provide implants. Larger urban centers staffed by nurses, midwives, and CHEWs and smaller rural health centers staffed by a nurse or midwife only will be eligible for inclusion.

A total of 60 facilities will be selected in each state from eligible facilities, yielding a total of 120 facilities. Where there are more than the required number of eligible facilities, simple random sampling will be used to select facilities for inclusion. At least 30 nurse or midwife-led facilities will be selected in each state to allow inclusion of 30 nurses and midwives in the study. The remaining 30 facilities will include as many CHEW-led facilities as possible. From each CHEW-led facility, 1 CHEW will be trained, and 1 nurse or midwife or 1 CHEW will be trained from each nurse or midwife-led facility. The providers trained will be the individuals responsible for providing FP services at their facility.

**Table 1:** Details of implant brands to be included in the study.

Product	Composition	Labeled duration of use	Format
Jadelle	150 mg levonorgestrel, 2 rods	5 years	2 rods, separate disposable trocar
Implanon Classic	68 mg etonorgestrel, 1 rod	3 years	1 rod preloaded in trocar

**Table 2:** Implant insertion adverse events to be recorded.

Description of adverse reaction		Day recorded relative to procedure
Anaphylactic reaction to the implant		0
Implant insertion unsuccessful on first or second attempt		0
Implant breaks		0
Palpitations resulting from the local anesthetic		0
Expulsion of implant		14
Paresthesia due to neural damage (numbness, tingling, tickling, pricking, or burning sensation at implant site)		14
Pain post procedure for >1 week and requires further outpatient observation and medical intervention		14
**Infection**		0
	Local redness swelling	14
	Discharge	14
	Fever	14
Scarring		14
Hematoma or bruising requiring medical intervention		0 and 14
Bleeding around the injection area		0 and 14
Other adverse reaction requiring medical treatment or resulting in long-term incapacity or fatality		0 and 14

### Intervention Implementation

The intervention will have 4 phases, which are discussed below.

#### Clinical Supervisor Training

A total of 12 clinical supervisors (qualified nurses or midwives with extensive experience of implant service provision) will be trained over 3 days. Training will cover how to train study providers to provide implants, clinical supervision of study providers, and the research study and data collection procedures.

#### Provider Training in Implant Provision and the Research

Clinical supervisors will train providers on counseling and on insertion and removal of Implanon Classic and Jadelle. Training will comprise classroom and clinic components and include a written test. Training will be based on the FMOH’s competency-based training package for LARCs. This involves working with providers at different levels to bring them up to the same level, and so the number of days of training can vary [24]. The implant clinical training materials, previously only used to train nurses and midwives, will be reviewed and adapted if necessary for CHEWs. Training will be free for providers.

All providers will be trained on the study protocol for managing adverse events, which is based on MSI’s adverse event standard operating procedures. The protocol details when to refer clients to a higher-level provider, particularly important for CHEWs who may be working in clinics without a higher-level provider, and adverse event support and reporting mechanisms. Any major adverse event must be reported immediately to the MSION clinical services manager, who will inform the study manager and the MSI medical development team in London within 24 hours. For this study, moderate adverse events will also be reported to the clinical services manager who will inform the study manager. Reportable adverse events are shown in Textbox 1. Other conditions are to be reported if the level of adversity is judged to be persistent or difficult to manage. The clinical services manager will be responsible for ensuring any adverse event reported is managed effectively.

MSION research staff will train providers on the research study, including participant recruitment and consent, data collection, and data storage. Full training (implant provision and research) is expected to take approximately 8 days.

#### Supervised Provision

After training, the trainee can provide implants to clients at their clinic only in the presence of a MSION clinical supervisor.

The trainee will encourage FP clients to attend on scheduled days when the supervisor is present. Clients will be counseled on the available methods, and if they choose an implant, they will be given the option of Jadelle or Implanon Classic. Supervision visits will take place every 2 weeks but exact timings will depend on the volume of implant clients. After 5 successful supervised insertions of each brand of implant, the trainee is accredited to insert implants without clinical supervision.

**Moderate and major adverse outcomes to be reported to ensure participant safety.**

Moderate level of adverse event

- Partial expulsion of implant, putting the woman at risk of pregnancy
- Pain at insertion site continues for more than 1 week and requires further outpatient observation and medical intervention
- Bleeding that does not stop and requires a transfer to receive medical care
- Infection that persists after 7 days of antibiotic treatment (may require implant removal)

Major level of adverse event

- Complete expulsion of the implant resulting in pregnancy
- Palpitations from the local anesthetic
- Bleeding that requires hospital care or results in long-term health impacts
- Infection that requires hospital care or results in long-term health impacts
- Paresthesia that requires hospital care or results in long-term health impacts
- Scarring that requires hospital care or results in long-term health impacts
- Anaphylactic response to the implant

MSION staff will conduct demand-generation activities in the local area to increase awareness of the range of contraceptive methods available, including implants, to ensure that trainees have sufficient clients. Activities may include in-facility awareness raising on special days such as antenatal care, immunization, and child welfare days, and recruiting designated locals to encourage potential clients in their communities to attend the nearest study facility for FP.

On the basis of MSION’s previous experience of conducting training and supervision of midwives, nurses, and doctors, it is expected that most providers will have been accredited for insertion by the end of their first supervision visit. Additional visits will be undertaken in cases where accreditation of insertion is not achieved within the prescheduled supervision visit.

#### Unsupervised Provision

After accreditation, participating providers will start to offer unsupervised implant services in their clinics and to recruit clients to the study. Participating providers will be invited to attend a monthly study meeting at the state level where they will receive study updates and tools and, if necessary, additional training. Providers will receive lunch and travel costs. Supervision visits will still occur but they will be less often following provider accreditation.

### Participant Recruitment and Eligibility

Women requesting an implant from a participating facility will be eligible to participate if they are aged between 18 and 49 years and are eligible to have the implant. On presenting at a participating clinic, each client will be provided with comprehensive FP counseling and offered all available FP methods (balanced counseling technique) [25]. Participating providers will invite all clients who request an implant, and meet the inclusion criteria, to participate in the study, using a study information sheet and consent form. If the client chooses an implant, she will be offered Jadelle or Implanon Classic, and the same service provider will do the insertion. Clients who want an implant but do not want to participate in the study will be advised of the nearest available facility providing this service.

Clients will be recruited until the sample size is reached (anticipated to be approximately 7 months). Participants who are asked to return for a 2-week follow-up visit will be reimbursed for their travel costs. Women who do not return after 14 days will be contacted by telephone (if they gave consent to be contacted and a phone number). In total, 3 attempts will be made to contact women by telephone before they are considered lost to follow-up. Those who are contacted by phone will be asked to attend the facility for the 14-day follow-up visit or, if unable, to answer questions over the telephone.

The number of clients who refuse to take part and who withdraw will be documented, along with the reason for refusal or withdrawal where given. If they agree, very basic sociodemographic data and the reason for refusal will be recorded.

All health care staff at participating facilities will be eligible for participation in semistructured in-depth interviews to assess acceptability of CHEW implant provision. A range of staff types (doctors, nurses, midwives, and CHEWs) will be approached by a senior research staff member and asked for their willingness to participate. Policy makers involved in FP will be contacted individually by researchers and asked to participate in an interview. All participants will be asked for their informed consent before the interview.

### Sample Size

#### Day of Insertion

Calculation of the number of participants required for the study is based on the primary outcome—the frequency of adverse events associated with insertion on the day of the procedure.

Studies documenting adverse events of implant insertion on the day of procedure have found them to be rare. For example, a randomized clinical trial of 2008 women found that 0.2% clients using Jadelle and 0.8% clients using Implanon Classic experienced complications at insertion [26].

To detect a difference in proportion of adverse events between the 2 study arms with a noninferiority limit of 0.5%, 80% power, and 95% confidence level on the day of implant insertion, we would require a sample size of 2462 in each study arm, giving a total sample size of 4924. To account for possible clustering, we will include a design effect of 1.5, increasing the sample size to 7386. To allow for incomplete records, we will increase this by 10% to a total sample size of 8125.

We estimate that each provider will receive approximately 10 insertion clients per month and that we may see a refusal rate of 20%. With a total of 120 providers, we expect to recruit 1200 clients per month. We would therefore need to recruit clients for approximately 7 months to achieve the required sample size for insertions.

#### By Day 14

We expect the number of adverse events observed by day 14 to be higher than that at day 0 as we may start to see infections. This reduces the required sample size needed to detect a difference between health care provider types. To detect a difference in proportion of adverse events between the 2 study arms with a noninferiority limit of 1%, 80% power, and 95% confidence level 14 days after implant insertion, we would require a sample size of 1225 in each study arm, giving a total sample size of 2450. To account for possible clustering, we will include a design effect of 1.5, making the size 3675. Assuming 20% loss to follow-up makes a total sample size of 4410. If we expect to recruit 1200 clients per month, it will take around 4 months to recruit the sample size we require.

All recruited clients will be asked to return to the inserting provider for a follow-up visit at 14 days post insertion until the sample size of 4410 is reached.

#### Quality and Acceptability of Implant Provision

To detect a difference in the quality and acceptability of implant provision between the 2 study arms, assuming that each group would score an average of 80% of quality and acceptability indicators being met, with a noninferiority limit of 10%, 80% power, and a significance level of 5%, we will need 198 observations in each group. Adjusting for 10% incompleteness, we need 220 in each group (440 total). Assuming a design effect of 1.8 requires a minimum of a total of 792 observed implant insertions. There will be approximately 10 providers per supervisor. So, assuming approximately 10% of insertions can be observed (and client acceptability measured through exit interviews), this equals 812 insertions, which is sufficient according to the sample size calculation.

#### Acceptability of Community Health Extension Worker Implant Provision to Health Staff, Facility Managers, and Policy Makers

Approximately 3 to 5 interviews with providers from each cadre will be conducted, until no new information or opinion is yielded from each subsequent interview. Views from the north and south, urban and more remote locations, and different ethnic and wealth contexts will be included; if views are already known, a mix of supporters and opponents of CHEW implant provision will be interviewed.

### Data Collection

#### Safety—Days 0 and 14

At the time of insertion, the provider will record baseline data about the implant insertion, the occurrence of adverse events, and the client’s background (including demographics, socioeconomic information, reproductive history, and reasons for getting an implant). At day 14 post insertion, the provider will ask the client about side effects or complications arising from the procedure.

To assess the ongoing safety of the study, any participant coming to the facility with complaints about her implant up to 30 days after the procedure will be asked a few questions by the provider about what the participant has experienced and her responses will be documented. Responses will be reviewed by project staff.

#### Quality of Implant Provision

Each provider will be visited by her clinical supervisor at 0 (accreditation), 1, 2, 3, and 6 months post training. During these 1 to 2 day long visits, all implant insertions will be observed, and data on quality recorded. Visits will be scheduled in advance and previsit FP demand-generation activities will be conducted by MSION and health care workers. The quality assessment tool includes clinical and nonclinical dimensions, including cleanliness, infection control, taking a client-focused approach, and being responsive to client needs. The tool comprises a checklist of 28 items, observed by the clinical supervisor. For each item, the supervisor will assess provider competence (ie, the provider knows the steps for the skills and can perform them correctly or needs further follow-up) and support to achieve competency (ie, the provider does not know the steps for the skills and does not perform them correctly). The number of items marked “competent” will be totaled to give the provider a score.

#### Acceptability of Implant Provision—Client Exit Interviews

During supervisor visits, clients participating in the study will be interviewed by the supervisor in private, before leaving the clinic. They will be asked to rate 7 aspects of service provision on a 5-point scale, with responses being totaled to give a satisfaction score. They will be asked if they would recommend the service to a friend.

Data collection points are summarized in Table 3.

**Table 3:** Summary of data collection procedures.

Timing of data collection	Who collects data, and how	Data to be collected	Sample size
At the time of procedure	Supervisor (if present), observes procedure	Quality of service provision	792
Immediately post implant procedure	Community health extension worker (CHEW), nurse or midwife who conducted procedure, observes client and asks her questions at clinic	Client background, record of procedure including complications at the time	8125
	Supervisor (if present), conducts exit interview with client	Client satisfaction, completeness of counseling information, critical information provision	792
2 weeks post procedure	CHEW, nurse or midwife who conducted procedure, asks client questions at clinic or by telephone	Complications following procedure	4410
Within 30 days of procedure	CHEW, nurse or midwife who conducted procedure, records client’s problems at clinic	Complications following procedure	All cases reported spontaneously at the facility

#### Acceptability of Community Health Extension Worker Implant Provision to Health Care Staff and Policy Makers

Interviews with staff members and policy makers will cover themes such as perception of confidence and competence in CHEW provision of insertions, support for policy change, perceptions of effectiveness of demand-generation activities, supervision levels, supply chain, and cost-effectiveness. Responses will be recorded on paper using shorthand notes with verbatim quotes as far as possible, and audio-recorded as a backup.

### Data Management

Client identifiers, such as name or phone number, will be recorded in a client register and will not be included on client data collection forms. The 2 forms will be stored separately and linked using a client identification number. Study documents will remain in locked cabinets in the provider’s office, if available, or in a secure location where medical records are secured.

Providers will keep the anonymized data collection forms for 30 days after insertion and will record any spontaneous client reports of adverse events over this 30-day period. After 30 days, the anonymized forms will be sent to MSION head office in Abuja. The client register will be kept at the clinic for the duration of the study. At the end of the study, client registers will be sent to MSION head office in Abuja and will be kept in a locked cabinet separate from data collection forms. Completed and anonymized exit surveys will be kept on the person of the clinical supervisor or researcher conducting the interview until the end of the visit, after which they will be sent to MSION in Abuja.

Data entry will be conducted by an independent consultant engaged by MSION. Paper forms will be checked for completeness and obvious errors by the data entry clerk when they arrive in Abuja, and queries will be checked by telephone with the provider, clinical supervisor, or researcher. The data entry clerk will double-enter all data into a password-protected electronic database, verify any discrepancies between the 2 data entries, and clean the data in preparation for safety monitoring analysis every 3 months. When all complication data have been collected, entered, and cleaned, the full dataset will be transmitted to the research team in MSI London for analysis.

The cleaned, anonymized electronic dataset will be kept by MSI London for a minimum of 5 years and will be made publicly available by request to Marie Stopes [27]. Paper forms will be kept securely by MSION in Abuja until 1 year after publication and then destroyed.

Audio recordings will be transcribed verbatim. The recordings will be stored in locked cabinets until they have been transferred to a computer, after which they will be destroyed.

### Data Analysis

Quantitative data will be analyzed using statistical software Stata, version 13 (Stata Corp. College Station, Texas). Complete case analysis will be conducted. For safety monitoring, complication rates by provider will be generated by the study manager in Abuja every 3 months, to identify any providers with unusually high complication rates. These data will be checked and response activities will be agreed at the 3 monthly technical advisory committee meetings to be held in Abuja, which will be attended by staff from MSION and MSI, and ministry of health officials.

Training and supervision data will be presented using descriptive statistics. Insertion adverse events of CHEWs will be compared for noninferiority against the complication rates of nurses and midwives. To assess the equivalence between the nurses and midwives and the CHEWs, the risk difference between the 2 provider types together with their 95% CI will be derived by use of a generalized estimating equation model to adjust for clustering. If the CI of the risk difference between the 2 groups falls within the predetermined margin of equivalence (–2% to 2%, which is equivalent of lower bound of one side 97.5% CI), the 2 types of service providers can be considered equivalent.

Sensitivity analysis will be designed to control for group assignment bias among the 2 states. They will be analyzed both separately and together to identify any differences in performance between CHEWs in the northern state compared with the southern state.

All quality and acceptability indicators will be weighted equally, except for those most critical (infection prevention, correct clinical technique, and advice on where to go in case of problems), which will be given a higher weight. The difference in mean quality and acceptability scores between CHEWs and midwives will be compared using a *t* test.

Analysis of qualitative data will be undertaken manually or using a qualitative analysis software such as N-Vivo. Quotations will be labeled by cadre of speaker, and a thematic analysis will be carried out. Transcripts will be read and reread. Extracts will be coded according to themes and subthemes that emerge from the data or that have been identified before the analysis such as those included in the in-depth interview guide. A selection of quotes representing different cadres and views will be used to write a report structured around these themes.

### Dissemination Policy

Findings will be shared with stakeholders through formal reports and presentations at local and national levels, and more broadly through peer-reviewed publications and international conference presentations. Authorship eligibility will be dependent on substantial contributions to planning, implementing, analyzing, or drafting of findings. The deidentified dataset will be made publicly available following the publication of study results.

## Results

In total, 12 LGAs were selected in Kaduna State and 23 in Ondo State. We trained 60 CHEWs and a total of 60 nurses and midwives (30 of each from each state) in September and October 2015, and recruitment took place between November 2015 and December 2016. Data cleaning is complete and analysis is being finalized. Results are expected in March 2018.

## Discussion

Besides resulting in 120 more health care staff qualified to provide contraceptive implants, this study will provide robust evidence on the safety, quality, and acceptability of contraceptive implant provision by CHEWs compared with nurses and midwives in Nigeria. This evidence may also serve to support future decision making about task-sharing implant services to community health workers in other countries. Strengths of the study include being able to compare CHEW provision with an existing standard of care (nurse and midwife provision). The study builds on existing training and supervision procedures, which will increase the sustainability and scalability of CHEW implant provision if the results are promising.

The main limitations of the study are the lack of randomization and that that providers will be assessing their own practices. Clients cannot be randomized because they usually access their local area clinic, and providers cannot be randomized because they work at either CHEW-led or at nurse- or midwife-led public clinics. Where both cadres work together, the national policy is to train 1 provider in each clinic. This could result in bias due to nonequivalence of the intervention and control groups (ie, if participating CHEWs tend to work in more remote clinics with poorer supply chain for infection prevention supplies than participating nurse and midwives). It is not feasible to arrange independent observation of all procedures; furthermore, observation itself may change practice. Bias will be assessed by comparing adverse event rates recorded by supervisors with those documented by providers themselves. Providers will be informed that this checking will take place.

Although both CHEWs and the nurses and midwives will be trained in and will conduct implant removals, there will be insufficient time during the study period to collect enough information on removals to make any noninferiority comparisons.

## Abbreviations

CHEW: community health extension worker
FMOH: Federal Ministry of Health of Nigeria
FP: family planning
IUD: intrauterine device
LARC: long-acting reversible contraceptive
LGA: local government area
MSI: Marie Stopes International
MSION: Marie Stopes International Organisation Nigeria
WHO: World Health Organization
